# Intensity modulated radiotherapy (IMRT) combined with concurrent but not adjuvant chemotherapy in primary nasopharyngeal cancer – a retrospective single center analysis

**DOI:** 10.1186/1748-717X-8-20

**Published:** 2013-01-24

**Authors:** Ladan Saleh-Ebrahimi, Felix Zwicker, Marc W Muenter, Marc Bischof, Katja Lindel, Juergen Debus, Peter E Huber, Falk Roeder

**Affiliations:** 1 Clinical Cooperation Unit Molecular and Radiation Oncology, German Cancer Research Center (DKFZ), Heidelberg, Germany; 2 Department of Radiation Oncology, University of Heidelberg, Heidelberg, Germany; 3 Department of Radiation Oncology, Katharinen-Hospital, Stuttgart, Germany

**Keywords:** Nasopharyngeal cancer, IMRT, Concurrent chemotherapy

## Abstract

**Background:**

We report our experience in 49 consecutive patients with nasopharyngeal carcinoma who were treated by Intensity-modulated radiation therapy (IMRT) combined with simultaneous but not adjuvant chemotherapy (CHT).

**Methods:**

The medical records of 49 patients with histologically proven primary nasopharygeal carcinoma treated with IMRT and concurrent platin-based CHT (predominantly cisplatin weekly) were retrospectively reviewed. The majority of patients showed advanced clinical stages (stage III/IV:72%) with undifferentiated histology (82%). IMRT was performed in step-and-shoot technique using an integrated boost concept in 84%. In this concept, the boost volume covered the primary tumor and involved nodes with doses of 66–70.4 Gy (single dose 2.2 Gy). Uninvolved regional nodal areas were covered with doses of 54–59.4 Gy (median single dose 1.8 Gy). At least one parotid gland was spared. None of the patients received adjuvant CHT.

**Results:**

The median follow-up for the entire cohort was 48 months. Radiation therapy was completed without interruption in all patients and 76% of the patients received at least 80% of the scheduled CHT. Four local recurrences have been observed, transferring into 1-, 3-, and 5-year Local Control (LC) rates of 98%, 90% and 90%. One patient developed an isolated regional nodal recurrence, resulting in 1-, 3-, and 5-year Regional Control (RC) rates of 98%. All locoregional failures were located inside the radiation fields. Distant metastases were found in six patients, transferring into 1-, 3, and 5-year Distant Control (DC) rates of 92%, 86% and 86%. Progression free survival (PFS) rates after 1, 3 and 5 years were 86%, 70% and 69% and 1-, 3- and 5-year Overall Survival (OS) rates were 96%, 82% and 79%. Acute toxicity ≥ grade III mainly consisted of dysphagia (32%), leukopenia (24%), stomatitis (16%), infection (8%) and nausea (8%). Severe late toxicity (grade III) was documented in 18% of the patients, mainly as xerostomia (10%).

**Conclusion:**

Concurrent chemoradiation without the addition of adjuvant chemotherapy cycles using IMRT with an integrated boost concept yielded good disease control and overall survival in patients suffering from primary nasopharyngeal cancer with acceptable acute side effects and limited rates of late toxicity.

## Background

Since the report of the Intergroup 0099 trial in 1998 by Al-Sarraf et al. [1], which showed a survival benefit with the addition of concurrent and adjuvant chemotherapy to radiation alone, and the confirmation of their results by several subsequent randomized trials and meta-analyses [2-7], concurrent chemoradiation has emerged as the standard of care at least for locally advanced stages of nasopharyngeal cancer. However, with regard to the still considerable rates of acute and late toxicities and the limited treatment compliance using this combination approach, some questions remain in terms of radiation technique, fractionation and especially value and timing of the adjuvant chemotherapy component. For example, intensity-modulated radiation therapy (IMRT) offers advantages in terms of target coverage or sparing of organs at risk compared to the frequently used conventional (2D-RT) or three-dimensional conformal (3D-RT) radiation techniques [8-10]. IMRT also simply allows a slightly accelerated fractionation in the boost areas (integrated boost). Encouraging results for IMRT with low toxicity have been reported in several single-center series [11-14]. Further on, many studies [1-3,15,16] investigating combined chemoradiation, also used adjuvant cycles of chemotherapy. This part of the treatment was associated with considerable toxicity, frequently not completed in a substantial proportion of patients, and a recently published randomised trial found no benefit for the addition of adjuvant chemotherapy cycles compared to concurrent chemoradiation alone [17]. Thus, some concerns have been raised about the need for additional adjuvant chemotherapy cycles, especially when modern radiotherapy techniques like IMRT are combined with simultaneous chemotherapy. Here we report our retrospective analysis of 49 consecutive patients over a 10-year period using a treatment approach consisting of IMRT with integrated boost combined with concurrent but not adjuvant platin-based chemotherapy. The data show that this approach is effective with limited toxicity.

## Methods

### Patient characteristics

We identified a total of 55 patients with primary nasopharyngeal cancer in the database of the German Cancer Research Center (DKFZ), who have been treated with IMRT and concurrent chemotherapy at our institution between 1999 and 2008. Data was obtained retrospectively by chart and radiation therapy documentation review. Six patients were excluded from analysis, because they had received also adjuvant chemotherapy. All patients suffered from histologically proven primary nasopharyngeal cancer without evidence for distant metastasis. Initial work-up included clinical and laboratory examinations, computed tomography (CT) and/or magnetic resonance imaging (MRI) of the head and neck region, endoscopy with histological confirmation, chest x-ray or CT, abdominal ultrasound or CT and bone scan for exclusion of distant metastases. The patients were staged according to the 6^th^ edition of the International union against cancer (UICC) TNM classification. Histological diagnosis was graded according to the world health organisation (WHO) classification for nasopharyngeal cancer. 17 patients had a surgical intervention before referral to our department, mainly as single lymph node exstirpation or selective neck dissection for diagnostic reasons. Two of them had incomplete local excisions. All patients had measurable gross disease at the beginning of concurrent chemoradiation. For detailed patient characteristics, see Table 1.

**Table 1:** Patient and treatment characteristics

	**n**	**%**
**Age**		
median	50	
range	18-71	
**Gender**		
male	37	76
female	12	24
**T stage**		
1	7	14
2a	5	10
2b	19	39
3	8	16
4	10	20
**N stage**		
0	10	20
1	12	24
2	21	43
3a	1	2
3b	5	10
**Clinical stage**		
I	2	4
IIa	1	2
IIb	11	22
III	21	43
IVa	8	16
IVb	6	12
**Histology**		
I	3	6
II	6	12
III	40	82
**Concurrent CHT**		
cis weekly	31	63
carbo weekly	2	4
cis/5-FU	11	22
carbo/5-FU	4	8
5-FU	1	2

Age [years], T, N and clinical stage according to 6^th^ edition of the International Union against Cancer (UICC) TNM classification, histology according to world health organisation (WHO) classification, Cis: Cisplatin, Carbo: Carboplatin, 5-FU: 5-Fluorouracil, n: number of patients,%: percentage of the entire cohort, CHT: concurrent chemotherapy.

### Radiation therapy

**Figure 1:**
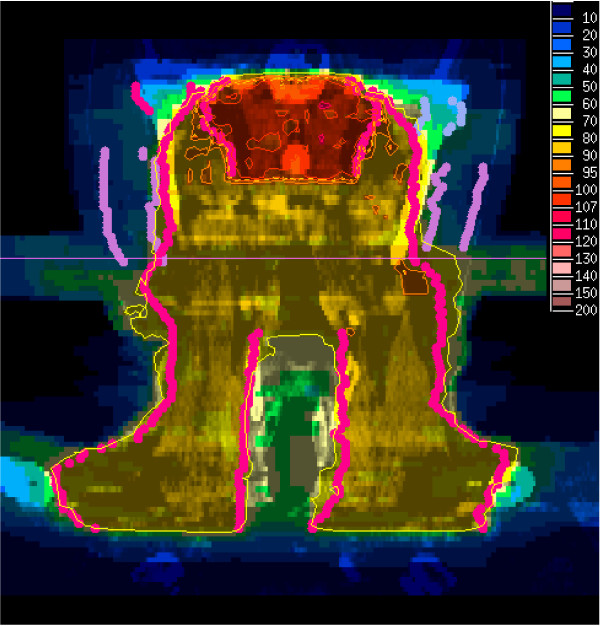
Example of dose distribution (frontal view, integrated boost concept, prescribed dose 70.4 Gy = 100% in 32 fractions, dotted line : 95% isodose, sparing of both parotid glands and larynx).

External beam radiotherapy (EBRT) was performed in intensity-modulated technique in all patients, using a step-and-shoot approach. The technique of IMRT used in our institution has been previously described [18,19]. Briefly, all patients were fixed in an individually manufactured precision head mask made of Scotch cast® (3 M, St.Paul, Minneapolis, MN) and a vacuum pillow for the body. With this immobilization system attached to the stereotactic base frame, contrast-enhanced CT- and MRI-images were performed with a slice thickness of at least 3 mm and fused based on the localizer-derived coordinate system. The gross tumor volume (GTV) was defined as the macroscopic tumor defined after correlative analysis of CT- and MRI-scans. In 59% of the patients additional GTVs were needed to cover involved nodes. For the clinical target volume (CTV) a margin of 0.5-1 cm was added manually to the GTV. A second CTV was defined including the bilateral uninvolved regional nodes (retro- and parapharyngeal nodes, cervical nodes Level II-V and supraclavicular nodes). A safety margin of 3–5 mm for the PTVs was added manually. Margins could be reduced in case of directly adjacent organs at risk. Inverse treatment-planning was performed using the KonRad and VIRTOUS software developed at the German Cancer Research Center (DKFZ). EBRT was delivered by a linear accelerator with 6 or 15 MV photons using an integrated motorized multileaf collimator (MLC) for the step-and-shoot technique automatically delivering the sequences. Since the introduction of a kV-CT on rails at our institution in 2002, all patients received image guidance (with the possibility for replanning if necessary) at least once a week. The total doses were prescribed to the median of the target volume and usually the 95% isodose surrounded the PTV. An integrated boost concept was used in the majority of patients (84%). According to this concept, the boost volume (primary tumor and involved nodes), was covered with doses of 66 to 70.4 Gy (median single dose 2.2 Gy) using 5 fractions per week. The uninvolved nodal regions were covered with doses of 54–59.4 Gy (median single dose 1.8 Gy). An example of a three dimensional dose distribution illustrating this concept is shown in Figure 1. At least one parotid gland was spared. For detailed information about the dose constraints see Table 2. In patients with sequential boost concept, conventional fractionation (single dose 1.8 to 2 Gy) was used.

**Table 2:** Dose constraints

**Dose constraints**	**Max [Gy]**	**Mean [Gy]**
brainstem	60 (surface)	54
temporal lobe°	60	
spinal cord	45	
optical nerves	54	
chiasma	54	
eye	50	25
lens	10	
brachial plexus*	60	
larynx		40
parotid gland’		26

*: in cases with involved nodes in the supraclavicular region, doses up to 66 Gy were tolerated in small regions, °: in case of T4 lesions, doses up to 66 Gy were tolerated in small regions, ‘: at least one parotid gland was restricted to a median dose of 26 Gy, Max : Maximum dose to the organ at risk, Mean : Mean dose to the organ at risk, Gy : Gray.

### Chemotherapy

All patients received chemotherapy concurrently to EBRT. The chemotherapy schedules varied over time, but were platin-based in 97% of the patients. The majority of the patients (63%) was scheduled for 6 cycles of weekly cisplatin at a dose of 40 mg per square meter body surface. Three patients had been treated with induction chemotherapy before referral to our department but none of the patients in this analysis received adjuvant chemotherapy. For detailed treatment characteristics see Table 1.

### Follow up

Regular follow up visits were performed at our institution or the referring center. At our institution, patients were scheduled for follow up visits every three months for the first two years, every 6 months for the three following years and annually thereafter. Each follow-up visit included at least clinical examination and CT or MRI of the head and neck region. In case of evidence for locoregional recurrence or distant spread, additional tests or imaging modalities were performed to confirm or exclude disease progression at the discretion of the treating physician. Missing data were completed by calling the patient or the treating physician.

### Definition of events

Local control (LC) was defined as absence of tumor (re)-growth in the region of the primary tumor. Regional control (RC) was defined as absence of tumor (re)-growth in the bilateral cervical nodal areas. Distant control (DC) was defined as absence of distant metastases. In patients without further assessment of local/regional control, for example after development of distant spread, the date of the last information about the local/regional status was used for calculation. Progression free survival (PFS) was defined as absence of disease progression at any site or death of any cause. Acute and late side effects were reported as documented in the patient charts. Acute toxicity was scored according to Common Toxicity Criteria version 3.0 (CTCAE V3.0) from the start of radiation therapy until 3 months of follow up. Late toxicity was scored according to CTCAE 3.0 thereafter until the end of follow-up. If multiple occurrence was documented, the most severe grade of a specific event was used for grading. Disease related functional impairments present prior to the start of chemoradiation were scored as toxicity only if worsening occurred. Xerostomia was scored as subjectively assessed by the patients and graded according to Radiation Therapy Oncology Group (RTOG)/European Organization for Research and Treatment (EORTC) radiation morbidity scoring criteria [20].

### Statistical methods

Time to event data was calculated from the first day of radiation treatment until the last follow up information or until death using the Kaplan-Meier method. Categorical variables were compared by Fisher’s exact test. Differences were considered statistically significant for a p-value of ≤ 0.05. The study is in compliance with the Declaration of Helsinki (Sixth Revision, 2008). Furthermore the study was approved by the Independent Ethics Committee of the Medical Faculty Heidelberg (Ref. Nr.: S-170/2012).

## Results

The median follow up for the entire cohort was 41 months and 48 months in survivors (range 6 to 122 months). Only three of the surviving patients had a follow-up interval of less than 2 years. EBRT was completed in all patients without treatment breaks >3 days. 76% of the patients received at least 80% of the scheduled chemotherapy.

### Local and regional control

**Figure 2:**
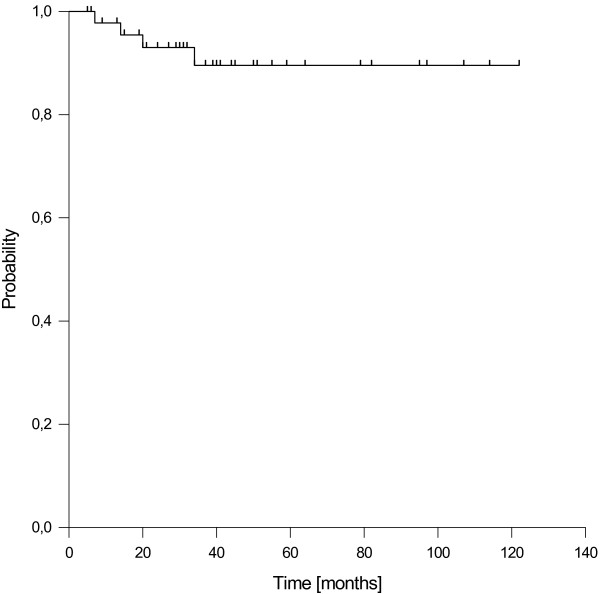
Local control.

We observed 4 local recurrences after 7, 14, 20 and 34 months of follow up. All local recurrences were located inside the boost areas. The resulting estimated 1-, 3- and 5-years local control (LC) rates were 98%, 90% and 90%, respectively (Figure 2). In the subgroup of patients with stage III/IV disease, the 5-year LC rate was 89%. Of the four patients with local recurrences, one was successfully salvaged by surgery (alive without evidence of disease at last follow up). One patient failed again after salvage surgery, and 2 patients were treated with palliative chemotherapy.

One additional patient suffered from an isolated nodal recurrence in the neck after 12 months, which was located inside the radiation fields. The resulting estimated 1-, 3- and 5-year regional control rates were 98%. The patient was successfully salvaged by neck dissection (alive without evidence of disease at last follow up).

### Distant control, progression-free survival and overall survival

**Figure 3:**
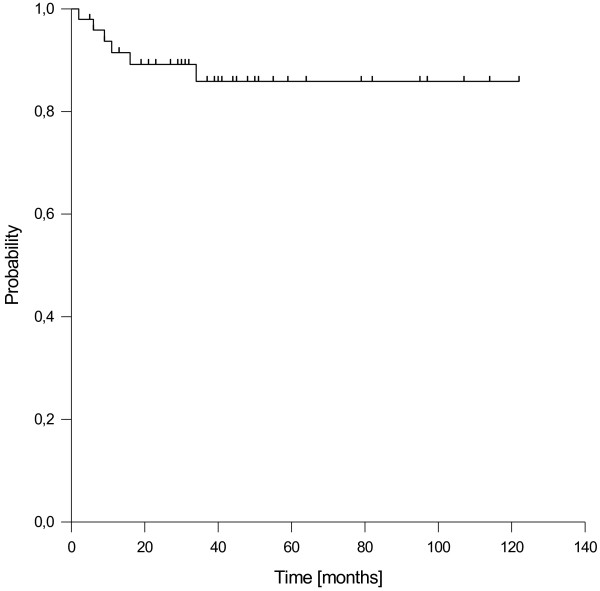
Distant control.

Distant metastases were observed in 6 patients after a median time of 10 months. Three patients developed bone metastases outside the head and neck region as first site of failure, one developed non-regional lymph node metastases and two patients suffered from visceral metastasis at multiple sites including lung and liver. The resulting estimated 1-, 3- and 5-year distant control rates were 92%, 86% and 86%, respectively (Figure 3). In the subgroup of patients with stage III/IV disease, a 5-year distant control rate of 80% was observed.

**Figure 4:**
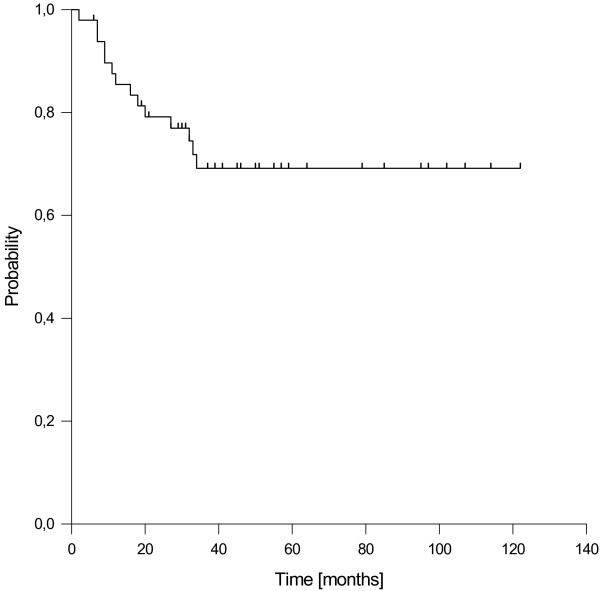
Progression-free survival.

Overall disease progression was found in 10 patients, 4 of them developed isolated locoregional failures and 5 isolated distant failures, whereas one patient suffered from a combined locoregional and distant failure. The resulting estimated 1-, 3-, and 5-year progression free survival rates were 86%, 70% and 69%, respectively (Figure 4). For stage III/IV patients, the 5-year progression free survival rate was 59%.

**Figure 5:**
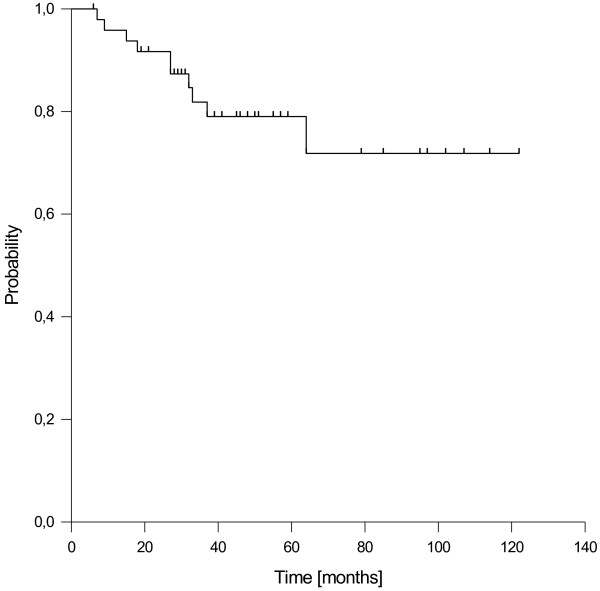
Overall survival.

Considering overall survival, we observed 10 deaths, including one patient who died due to advanced testicular cancer and one patient who died due to a non-treatment related sepsis 9 months after the end of concurrent chemoradiation. The resulting estimated 1-, 3-, and 5-year overall survival rates were 96%, 82% and 79%, respectively (Figure 5). For stage III/IV patients the 5-year overall survival rate was 73%.

### Functional impairments prior to chemoradiation treatment

Beside treatment-related toxicity, some patients showed already disease-related alterations of physiological functions at diagnosis or prior to chemoradiation treatment. These were mainly caused by compression or direct invasion of the primary tumor into adjacent structures resulting for example in middle ear effusion with hearing loss or cranial nerve palsy. For detailed information about functional impairments prior to chemoradiation see Table 3.

**Table 3:** Functional impairments prior to chemoradiation treatment

**Functional impairment**	**n**	**%**
middle ear effusion	22	45
hearing loss	18	37
t-tube placement	13	27
CN impairment (III, IV, VI)*	9	18
CN impairment (other)	9	18
nasal obstruction	7	14
headache	5	10
impaired vision°	4	8
epistaxis	4	8
dysphagia	3	6
tinnitus	2	4
impaired smell	2	4
impaired taste	2	4

CN: cranial nerve, *: including symptoms caused by impairment of the corresponding muscles, °: other than impairment of motility (see CN impairment), t-tube : inserted into tympanic membrane to restore ventilation in case of middle ear effusion.

### Acute toxicity

Mild to moderate acute toxicities were documented in the majority of patients, mainly as hematological toxicity or mucosa-related side effects. The main severe hematological side effect (≥ grade 3) was leucopenia. The main severe non-hematological side effect (≥ grade 3) was dysphagia (28 patients, 57%). However, 18 of these patients had received a prophylactical placement of a percutaneous feeding tube (PFT), which complicated the scoring of acute dysphagia because of the tendency to use an already placed PFT for at least parts of the nutritional support regardless from the real extent of their need. Considering only the 31 patients without prophylactical placement of a PFT, severe dysphagia was documented in 10 pts (32%) only. For detailed information about severe acute side effects see Table 4.

**Table 4:** Severe acute toxicity

**Severe acute side effects grade ≥ 3**	**n**	**%**
non-hematological		
dysphagia		
including proph. PFT	28	57
excluding proph. PFT	10	32
stomatitis	8	16
nausea	4	8
weight loss	3	6
nephropathy	1	2
hematological		
leucopenia	12	24
infection (including FUO)	8	16
thrombopenia	1	2
anemia	1	2

(≥ grade III) n: number of patients,%: percentage, proph.: prophylactical, PFT: percutaneous feeding tube, FUO: fever of unknown origin, some patients developed more than one toxicity.

### Late toxicity

The main documented late toxicity was xerostomia (see Table 5). Severity of xerostomia tended to be higher in patients with sparing of one parotid vs. patients where both glands were attempted to be spared. In these patients, the combined rate of grade 2 and 3 xerostomia was 37%, compared to 21% in patients with sparing of both glands, but this difference did not reach statistical significance. The remaining documented late toxicities are summarized in Table 6. The patient with severe trismus was the only patient with the need for long term support with a percutaneous feeding tube. Both patients who developed severe hearing loss (requiring hearing aids) had already suffered from reduced hearing function prior to chemoradiation. One patient with hyposmia prior to chemoradiation developed complete loss of smell and taste. No temporal lobe necrosis has been documented.

**Table 5:** Xerostomia

**Xerostomia**	**n**	**%**
grade 1	27	55
grade 2	10	20
grade 3	5	10
**Xerostomia**	**parotid glands spared**	
	one	both
grade 0-1	19	15
grade 2-3	11	4

According to Radiation Therapy Oncology Group (RTOG) criteria as subjectively assessed by the patients.

**Table 6:** Late toxicity

**Late toxicity**	**All grades (%)**	**Grade ≥ 3 (%)**
hearing loss	10	4
mucosal reaction	14	4
trismus	4	2
loss of taste	29	na
loss of smell	14	na
skin reaction	18	0
dysphagia	12	0
lymph edema	10	0
hoarseness	6	0
dry eye	6	0
other°	20	2

Excluding Xerostomia, scored according to CTCAE 3.0, na : not applicable (maximum score according to CTCAE 3.0: grade II), °: includes one patient (2%) with permanently reduced but stable renal function scored as grade 3 toxicity, some patients developed more than one toxicity.

## Discussion

Here we show in 49 consecutive patients over a 10 year period suffering from primary nasopharyngeal cancer, that encouraging local control (5-year LC 90%) and overall survival (5-year OS 79%) rates can be achieved with acceptable acute and limited late toxicities using IMRT combined with concurrent but not adjuvant platin-based chemotherapy. Despite the limitations of retrospective analyses of single institutions our results are in good accordance with other IMRT-series [11-14,21-23] describing similar results regarding outcome and toxicity (see Table 7).

**Table 7:** IMxRT series

**IMRT Series**								
**Author**	Lee et al.	Wolden et al.	Kam et al.	Kwong et al.	Tham et al.	Lee et al.	Peponi et al.	**Own data**
**Year**	2002	2006	2004	2006	2009	2009	2010	**2012**
**Institution**	UCSF	MSKCC	PWH	QMH	NCC	RTOG0225	USZ	**DKFZ**
**Region**	USA	USA	Hongkong	Hongkong	Singapore	USA	Switzerland	**Germany**
**n**	67	74	63	50	195	68	39	**49**
**f/u**	31	35	29	25	27	31	30	**48**
**Stage III/IV**	70%	77%	57%	100%	56%	59%	85%	**72%**
**sim. CHT**	75%	93%	25%	68%	57%	83%	97%	**100%**
**adj. CHT**	75%	93%	0%	68%	35%	83%	97%	**0%**
**TD**	65-70	70	66	76	70	70	66-70	**66-70,4**
**SD**	2,12-2,25	2,34 o. CB	2	2,17	2,0-2,12	2,12	2,0-2,2	**2,2**
**add. Boosts**	Br 40%	CB 80%	Br/3D 56%	none	Br 10%	none	none	**none**
**LC (year)**	96% (4y)	91% (3y)	92% (3y)	96% (2y)	90% (3y)	93% (2y)	86% (3y)	**90% (5y)**
**RC (year)**	98% (4y)	93% (3y)	98% (3y)	n.r.	n.r.	91% (2y)	89% (3y)	**98% (5y)**
**DC (year)**	66% (4y)	78% (3y)	79% (3y)	94% (2y)	89% (3y)	85% (2y)	85% (3y)	**86% (5y)**
**OS (year)**	88% (4y)	83% (3y)	90% (3y)	92% (2y)	94% (3y)	80% (2y)	85% (3y)	**79% (5y)**

n: number of patients, f/u: median follow up (months), sim.: simultaneous, adj.: adjuvant, CHT: chemotherapy, TD: total dose, SD: single dose, add.: additional, LC: local control, RC: regional control, DC: distant control, OS: overall survival, y: year, Br: Brachytherapy boost, CB: concomitant boost, 3D: 3d-confomal boost, nr : not reported, UCSF: University of California San Francisco [12], MSKCC: Memorial Sloan-Kettering Cancer Center [11], PWH: Prince of Wales Hospital [14], QMH: Queen Mary Hospital [13], NCC: National Cancer Center [21], RTOG: Radiation Therapy Oncology Group [22], USZ: Universitätsspital Zürich (University Hospital Zuerich) [23], DKFZ: Deutsches Krebsforschungszentrum (German Cancer Research Center).

IMRT has been shown to result in dosimetric advantages compared to other radiation techniques in nasopharyngeal cancer cases [8-10], which theoretically should lead to reduced late toxicity, especially in terms of xerostomia. Accordingly, we observed limited rates of xerostomia (combined grade 2/3 : 30%) in our analysis, which seemed to be further reduced in patients with sparing of both parotid glands, although this difference was not statistically different. It should be noted that scoring of xerostomia is controversial, especially in retrospective series and comparisons of different reports are compromised by applying different assessment strategies and grading scales. This may have contributed to the wide range of reported grade 2/3 xerostomia (21%-57%) even considering only IMRT series [11,12,14,22,23]. Scoring of xerostomia can be further complicated due to its possible changes over time [12,14,22-24]. Nevertheless, the present results are in good accordance with prior reports of our group, which showed effective protection of parotid function measured by quantitative pertechnetat scintigraphy in head and neck cancer patients treated with IMRT compared to other radiation techniques [25,26]. Moreover, Kam et al. [24] observed a significant reduction of observer rated xerostomia paralleled by a significant increase in stimulated parotid and whole saliva flow rate in a meticulously performed randomized comparison of IMRT vs. 2D-RT, although there was no significant difference in patient reported outcomes between the two arms. In contrast, Pow et al. [27] found that the reduction of xerostomia also transferred into improved quality of life in a similar comparison. In summary, there is growing evidence that IMRT can lead to decreased rates of severe xerostomia compared to other radiation techniques by sparing dose to one or both parotid glands, although careful patient selection for sparing of both parotid glands seems mandatory and the attempt has to be weighed against target coverage as highlighted by reports on intraparotideal recurrences [28].

IMRT also offers the opportunity to increase the fractionation dose inside the boost area using an integrated boost concept, while keeping the single dose below 2 Gy in most organs at risk at the same time. Therefore many investigators [12,13,21-23] used at least slightly increased single doses (2.12-2.25 Gy) inside the boost area without markedly increased total doses. Consistent with our analysis, in which the majority of patients received a single dose of 2.2 Gy in the boost area up to a total dose of 66–70.4 Gy, those regimens were generally well tolerated. In contrast, Bakst et al. [29] reported excessive toxicity in terms of temporal lobe necrosis (3 patients, 12%) in a prospective trial of 25 patients treated with single doses of 2.34 Gy in the boost area to a total dose of 70.2 Gy. Two of the three patients suffered from T4 tumors and the region of temporal lobe necrosis was located inside the boost PTV or directly adjacent to it. Because no temporal lobe necrosis had been observed in a prior series of patients treated at the same institution with a single dose of 2.12 Gy, they concluded that this regimen was not safe. However, integrated boost concepts appear to result in superior dose distributions compared to sequential IMRT boost concepts according to a planning study [30], especially regarding the dose to most other organs at risk. Taken together single doses of up to 2.2 Gy inside the boost areas appear to be safe. However, caution is mandatory due to the extreme narrow therapeutic margin and patients with invasion of intracranial structures might be not ideal candidates for this strategy. For those patients, charged particles such as protons or carbon ions could be beneficial because of their dosimetric advantages. A recent planning comparison from our institution has shown improved target coverage and pronounced sparing of organs for a 3-field spot scanning intensity modulated proton technique vs. 9-field step and shoot photon IMRT [31].

Despite the retrospective nature and limited patient number of our analysis, the outcome of our patients with locally advanced nasopharyngeal cancer treated with IMRT and concurrent but not adjuvant chemotherapy is comparable to the results published by other groups using similar approaches with the addition of adjuvant chemotherapy cycles [11-13,21-23]. Irrespective of the advances in radiation therapy technique, the addition of chemotherapy had been a major step towards improved overall outcome in locoregionally advanced nasopharyngeal cancer as shown by several randomized trials and meta-analyses [1-5,7,16]. While little controversy exists about the benefit of chemotherapy applied concurrently to radiation therapy, the value of additional adjuvant chemotherapy has been debated extensively mainly due to limited treatment compliance and substantial toxicity in several trials. For example, 45% of the patients in the INT 0099 trial [1] did not receive all planned adjuvant chemotherapy cycles and about one third did not receive any. Moreover, in the remaining patients receiving adjuvant chemotherapy 52% had grade 3/4 toxicities. Similar findings have been reported by Wee et al. [2] and a limited compliance has been observed also in RTOG 0225 [22], which used IMRT as radiation therapy technique. Considering oncological outcome, three randomized trials have been performed to examine the benefit of adjuvant chemotherapy mainly compared to radiation alone [6,32,33]. None of them showed a benefit in terms of event-free or overall survival and two of them even failed to show a benefit in distant control [6,32]. Moreover, the meta-analysis by Baujat et al. [7], which in fact showed a survival benefit in favour of adding chemotherapy to radiation therapy, concluded that this benefit was attributable to the concomitant rather than to the adjuvant phase. Conversely, a combined analysis [34] of 441 patients from two randomized trials (NPC 9901 and NPC 9902), who had received radiation therapy as sole treatment or combined with concurrent and adjuvant chemotherapy, found a significant beneficial impact of chemotherapy on distant control which was attributed to the adjuvant phase according to the subsequent multivariate analysis [34]. According to this analysis, patients developed significantly less distant metastasis if they received 3 or more cycles of adjuvant chemotherapy compared to those with 0–1 cycles, indicating a value for adjuvant chemotherapy if adequate doses can be achieved [34]. Finally, the issue of adjuvant chemotherapy after chemoradiation has been addressed by a recent prospective randomized multicenter trial [17]. In this trial, more than 500 patients with non-metastatic locally advanced nasopharyngeal cancer were assigned to either concurrent chemoradiation using weekly Cisplatin followed by three cycles of adjuvant chemotherapy with cisplatin/5-fluorouracil or to concurrent chemoradiation alone. Primary endpoint was failure-free survival. Radiotherapy was given with single doses of 2–2.27 Gy in 5 fractions per week to a total dose of ≥66 Gy to the primary tumor and 60–66 Gy to the involved neck areas. Different radiation techniques were permitted including IMRT. Compliance to radiation therapy and concurrent chemotherapy was similar in both arms, but 18% of the patients scheduled for the adjuvant phase did not receive adjuvant chemotherapy at all, 37% did not receive all three cycles, 69% of the patients had treatment delays and 42% experienced grade 3–4 toxicity during the adjuvant phase. With a median follow up of 38 months, the estimated 2-year rates of failure free survival did not differ significantly between the arms, nor did the estimated 2-year rates of locoregional failure free survival, distant-failure free survival or overall survival. Treatment group was also not a significant predictive factor for any of the mentioned endpoints in the multivariable analysis. The authors concluded, that adding three cycles of adjuvant chemotherapy did not improve outcome compared to chemoradiation alone. They discussed that acute toxic effects during concurrent chemoradiation decreased the tolerance of the patients to adjuvant chemotherapy causing the limited treatment compliance.

In summary, treatment recommendations towards the benefit of adjuvant chemotherapy after concurrent chemoradiation remain controversial. However, data from numerous phase II trials indicate, that the benefit of additional chemotherapy might be exploitable by a different timing [35] and/or the use of more potent regimens including taxanes [17]. High rates of treatment compliance [36], with excellent rates of distant control and overall survival [35] have been reported, using a neoadjuvant approach with upfront chemotherapy followed by concurrent chemoradiation. Given the potential advantages of a neoadjuvant approach [37], this strategy seems promising and is currently evaluated in several phase III trials.

## Conclusion

IMRT with concurrent but not adjuvant platin-based chemotherapy resulted in encouraging rates of local and distant control, progression-free and overall survival with acceptable rates of acute and limited rates of late toxicity in patients with nasopharyngeal cancer. Using an integrated boost concept with single doses of 2.2 Gy in the boost areas appears to be safe and effective. Our findings of a single institution in consecutive patients treated from 1999 to 2008 are in good accordance with other series with or without adjuvant chemotherapy. Based on the available evidence, the value of additional adjuvant chemotherapy appears to be limited. Future directions might include neoadjuvant chemotherapy and potentially the introduction of charged particles, a strategy which needs to be investigated in controlled trials.

## Competing interests

The authors declare that they have no conflicts of interest.

## Authors’ contributions

LSE performed main parts of data acquisition, data analysis, literature review and manuscript draft. FZ, MWM, MB and KL participated in data acquisition, data analysis, literature review and patient treatment. JD and PEH participated in planning of the analysis, patient treatment and revised the manuscript critically. FR planned the analysis, participated in data acquisition, data analysis, literature review, patient treatment, manuscript draft and revised the manuscript critically. All authors read and approved the final manuscript.
